# Developing a Family-Centered Care Model in the Neonatal Intensive Care Unit (NICU): A New Vision to Manage Healthcare

**DOI:** 10.3390/ijerph17197197

**Published:** 2020-10-01

**Authors:** Sagrario Gómez-Cantarino, Inmaculada García-Valdivieso, Eva Moncunill-Martínez, Benito Yáñez-Araque, M. Idoia Ugarte Gurrutxaga

**Affiliations:** 1 Department of Nursing, Physical and Occupational Therapy University of Castilla-La Mancha, 45071 Campus Toledo, Spain; Sagrario.Gomez@uclm.es (S.G.-C.); maria.ugarte@uclm.es (M.I.U.G.); 2 Mostoles University Hospital (HMOS), Madrid Health Service (SERMAS), 28935 Mostoles, Spain; 3 Toledo Hospital Complex (CHT), Neonatal and Pediatric Oncology Unit, Castilla-La Mancha Health Service (SESCAM), Theoretical collaborator University of Castilla-La Mancha, 45071 Campus Toledo, Spain; mevamoncunill@hotmail.es; 4 Department of Physical Activity and Sports Sciences, University of Castilla-La Mancha, 45071 Campus Toledo, Spain; benito.yanez@uclm.es

**Keywords:** infant, newborn, family, empowerment, child development, critical care

## Abstract

Family-centered care (FCC) currently takes a greater role in health care, due to the increasing empowerment parents experience. Within neonatal intensive care units (NICUs), family participation has an impact on the humanized care of the preterm newborn (PN). This integrative review conducted according to Whittemore and Knafl investigated current knowledge of the FCC model and its application in PN care in specific units. The data were collected from PubMed, Cochrane, CINHAL, Scopus, and Google Scholar. A total of 45 articles were used, of which 13 were selected which met inclusion criteria. Their methodological quality was evaluated using the mixed method appraisal tool (MMAT), and after they were analyzed and grouped into four thematic blocks: (1) parental participation; (2) health parental training; (3) benefits of family empowerment; and (4) humanized care. The results revealed that FCCs promote the integration of health equipment and family. In addition, parents become the primary caregivers. The benefits of the family–PN binomial enable an earlier hospital discharge. Humanized care involves an ethical approach, improving health care. Changes are still needed by health managers to adapt health services to the needs of the family and PNs.

## 1. Introduction

The World Health Organization (WHO) defines the preterm newborn (PN) as an infant whose birth occurs before 37 weeks of gestational age are completed [1,2,3]. Due to disruption in the gestation process, PNs may experience a high degree of organic immaturity, a condition that sometimes creates the need for an infant to be transferred to a neonatal intensive care unit (NICU). In these units, the environment is characterized by the presence of a large number of harmful stimuli, such as excessive manipulation and even artificial light, which can affect the proper neurological development of these preterms [4,5,6]. In addition, there are cases of alterations related to emotional development in PNs, due to the loss of the mother–PN link [7]. This aspect may be related to the presence divided into time slots of the family, and the exclusion of the family in the conduct of basic care, by the health team in the NICU [7,8].

Beginning in 1970, a transformation in neonatal care management began in the NICU [8], when the “family-centered care” (FCC) model was implemented. In this model, the concept of humanization became very important [9,10]. This approach emphasizes the importance of achieving adequate sensorineural and emotional development in the neonate by optimizing both the macro-environment (lighting and sound) and the microenvironment (correct postural control, proper pain management, and minimal manipulations) [8,11]. All these changes involve a transformation inside these units, allowing greater similarity to the maternal uterus [12,13,14]. The application of FCCs enhances the involvement of parents in care, favoring family attachment by integrating them into the health team and, in turn, empowering them to achieve more humanized attention [7].

In 1986, Dr. Heidelise Als developed the “newborn individualized developmental care and assessment program” (NIDCAP) method [11]. This is based on individualizing, observing, and assessing the state of development in which the PN is located. It also assesses its ability to cope with stress before, during, and after care [15]. The NIDCAP method considers the preterm together with the family, a single unit to care for, involving parents in health care [8]. Because the NIDCAP method allows knowing the mature status of each newborn, healthcare professionals in these units should know and be trained in NIDCAP [16,17]. 

Currently, child hospitalization is associated with the presence of the parents, uninterrupted throughout a period of 24 h. However, in the context of intensive care, this situation is challenging, due to the high complexity of the care offered and the reduced family visitation hours still in existence in some NICUs [17,18]. In this area, FCCs involve parents in the participation and administration of their participation, thus contributing both to their training and to the establishment of the subsidiary–mother bond [12,19]. In order for the family to participate in such care, it is necessary to promote their empowerment through the establishment of an effective therapeutic relationship with the health team [20,21]. The role of nurses, because of their continuous presence in the NICU, progresses from that of a care provider to that of an educator in health and accompaniment in the training process of the main caregivers [15,16]. Another key element is to create a trusted therapeutic relationship where parents feel safe and confident enough to provide the PN with basic quality care [7,8]. The role of the principal caregiver in this unit will facilitate the departure from the hospital to the home after hospital discharge [10].

Family involvement is one of the health pillars underpinning FCCs [7,8]. According to the studies consulted [22,23], it is the parents themselves who need to actively participate in daily routines. They need to be heard, understood, and recognized according to the true role they play [23]. They should be guided by the health team in this process, so that they overcome the obstacles caused by early hospitalization of the PN [17]. Nursing plays an important role in listening to the demands, concerns, and fears of the family, planning care, attending to their needs and providing the necessary support, with the aim of making the NICU experience as stress-free as possible [16].

The paradigm shift toward more humanized care and the implementation of FCCs, which promote the constant presence of parents 24 h a day, make it possible to establish a partnership between caregivers and the health team [9,10]. Applying this methodology results in a decrease in anxiety, stress levels, and an increase in the sense of safety and control in parents. For the neonate, it is a better adaptation to the extrauterine environment, favoring the regulation of vital signs (heart rate, respiratory, and temperature) and greater weight gain. The continued presence of parents facilitates and promotes breastfeeding [14,18]. The improvement in the regulation of vital signs and the empowerment of families (parents, grandparents, uncles/aunts, and legal custodians) represents an increase in early highs, relative to the NICU where this model is implanted, decreasing the anxiety of the main caregivers and facilitating the transition to the home. At present, 21 centers are established internationally: 9 in the United States (Boston, Oklahoma, San Francisco, Carolina, Colorado, St Luke’s, Illinois, Cincinnati, Phoenix), 11 in Europe (Sweden, France, Netherlands, Belgium, Norway, Spain, Italy, Denmark, Portugal, and Germany), and 1 in South America (Argentina). Another 6 centers are being developed in Canada, Israel, Japan, and Southern Europe. Specifically in Spain, these units have been available since 2005 in Madrid (Hospital 12 de Octubre), and Barcelona (Hospital Maternal Infantil Vall d’Hebron) [11,13,24].

The main objective of this work is to conduct an integrative review to examine the extent to which published research articles on parental empowerment show active participation in NICUs from the FCC perspective. The secondary objectives were: (1) to determine the parents’ perspective on their involvement in care management; (2) to identify the training that parents acquire, and which is offered by health professionals; (3) to assess whether family involvement in these units contributes to meeting the basic needs of the preterm newborn; and (4) to understand and improve the humanization of care within the NICU.

## 2. Materials and Methods

### 2.1. Study Design 

An integrative review has been carried out, using the Whittemore and Knafl methodology, which uses 5 stages developed in this review: (1) The problem was identified by S.G.-C. and I.G.-V.; (2) the search for relevant literature published from 2009 to 2020 was carried out by the entire research team (S.G.-C., I.G.-V., E.M.-M., B.Y.-A., and I.U.-G.); (3) the selected data were evaluated using the mixed method appraisal tool (MMAT) by S.G.-C., I.G.-V., and B.Y.-A.; (4) the data were analyzed using inductive content analysis by E.M.-M., I.G.-V., and I.U.-G.; (5) the results were synthesized in the tables by S.G.-C., I.G.-V., and B.Y.-A. and reviewed by E.M.-M. and I.U.-G. [25].

In this type of research, various types of research are jointly used, including qualitative design, quantitative, or even mixed [26]. A question that provides a better understanding of the phenomenon was developed to be studied. For nursing, a greater potential is represented in the development of clinical practice, as it allows creating and reviewing scientific evidence [25,27].

The initial stage of an integrative review study is to identify a problem observed in health care and to propose an objective; in this case, the general purpose of this review was to examine the empowerment of parents by the health team, in the management of care from the perspective of the FCCs, in the context of having a child in the NICU. Therefore, does the application of FCCs in health care contribute to family empowerment in NICUs?

### 2.2. Search Strategy

Following the methodology of Whittemore and Knafl [25], the second stage included developing a search strategy in which the following databases were used: PubMed, Cochrane, CINHAL (Cumulated Index of Nursing Allied Literature Health), Medline, Scopus, and Google Scholar. For the search formula, MeSH terms were used in order to perform a more thorough inquiry. In addition, word blending was used when appropriate to reflect the syntax and search rules common to individual databases (Table 1).

The inclusion criteria set for the selection of articles were: (1) publication period from April 2009 to May 2020, due to the long-distance topic (years 80–90), but also the fact that interest and scientific production has increased in recent years; (2) design of the studies included in an integrative review: quantitative, qualitative, and mixed; (3) the issue related to the empowerment and participation of parents in the care of the PN within the NICU; (4) peer-reviewed articles. The search for literature yielded research on 4 continents, with no studies found in Africa. Specifically in the Americas, studies developed in Canada, Mexico, Brazil, Chile, Argentina, and Colombia were selected. In Europe, 5 studies were found, 2 of which were conducted in Sweden, another 2 in Italy, and 1 in the Netherlands. A singular study is provided from Asia, specifically in India. In the Oceania region, one study was found, which was conducted in Australia. In the documents found, the Anglo-Saxon language predominates, although it is also worth noting those related to the Portuguese language.

The exclusion criteria established were: (1) date prior to the pre-established limits; (2) articles related to the vision of the healthcare professional, as this research focuses on the empowerment of parents and the family; (3) typologies of narrative, bibliographic, systematic, and meta-analysis reviews, because they are not covered by integrative reviews.

Further research was carried out, consulting the bibliography of each of the revised articles, in order to deepen the search for information in relation to the objective of study.

**Figure 1:**
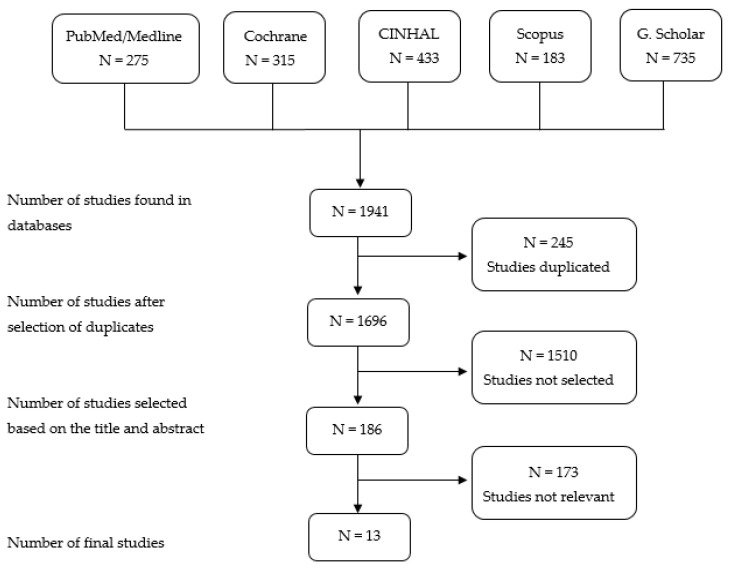
Search results and study selection. Source: Own elaboration of the authors.

A total of 1941 articles were collected, of which, after removing the duplicates, 1696 were obtained. After reading the title and summary of the articles by the research team, 1510 were discarded for not specifically addressing the study objective. Another 173 were also deleted, as these were not research-relevant studies, as they did not achieve the perspective of the thematic blocks reviewed in this study. Finally, a figure of 13 items was reached, with sample sizes ranging from 8 to 141 families (mothers and fathers) (Figure 1).

### 2.3. Quality Review and Evaluation Process

**Figure 2:**
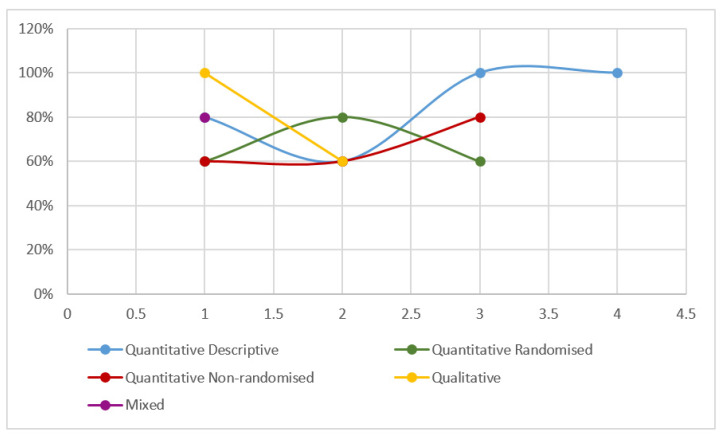
Methodological quality level (mixed method appraisal tool—MMAT), 2018. Source: Elaboration made by the authors, from Hong et al., 2018.

In an integrative review, the evaluation of the methodological quality of the studies [25] is not essential. However, to add scientific rigor to this research, a critical appraisal tool, designed for systematic reviews that include qualitative, quantitative, and mixed studies, was used in this research, called MMAT [28]. The MMAT was developed in 2006, revised in 2011, and its latest version was published in 2018, which has been used in this article [25,28]. Using this tool, researchers S.G.-C., I.G.-V., and B.Y.-A. scored the 13 articles analyzed, reviewing each of the five sections included in the MMAT (consisting of five questions) with 20%, 40%, 60%, 80%, and 100%, obtaining the level of methodological quality. Previously, the studies had been grouped by researchers E.M.-M., I.G.-V., and I.U.-G. into categories, resulting in a total of 4 quantitative descriptive studies; 3 quantitative randomized studies; 3 quantitative non-randomized studies; 2 qualitative studies; and 1 mixed study (Figure 2).

As noted, three of the evaluated articles have a 100% qualified methodological quality, as these investigations answered in the affirmative the five questions raised within the blocks that make up the MMAT. They correspond to two descriptive quantitative articles [29,30] and one qualitative study [31]. Regarding research that reached a score of 80%, there are studies which obtained only a negative rating in the evaluation. Among these is a descriptive quantitative study [32], another non-random quantitative study [33], a random quantitative study [34], as well as a mixed study [35]. Among the 60% score studies are studies that obtained only a positive rating in three of the five issues in each of the paragraphs, finding a descriptive study [36], two random quantitative studies [22,37], two non-random quantitative studies [38,39], and a qualitative study [40].

### 2.4. Data Analysis

Within the fourth methodological stage of the integrative review [25], for the extraction and summary of the data of the selected articles, a structured template was used as a table. This included the following 9 elements: author, year, and country; studio design; purpose; sample characteristics; main variables; methodological quality level; and results and limitations. Groups were assigned to the articles according to the type of design of the study for the presentation: quantitative, qualitative, and mixed. In addition, they were sorted chronologically by publication date, from the oldest to the most recent. In the case of matching multiple articles in the same year, they were sorted alphabetically according to the first author’s last name. Studies were not excluded based on the degree of rigor, since the objective of the integrative review was to synthesize the results of the revised research, to promote a change in the construction of knowledge for the nursing profession.

## 3. Results

The findings found in the 13 selected studies were classified into 4 common thematic blocks for further development. The main story lines focus on: (1) knowing the views and perspective of parents on their participation in PN care at the NICU; (2) exploring the training process experienced by parents by the health team; (3) determining the beneficial effects of incorporating the family into the conduct of PN care within the NICU; and (4) strengthening the humanization of care in the NICU. 

When the studies covered 2 areas of interest from those described above, they were included in both categories, to proceed with the extraction of all the results (Table 2).

### 3.1. Perspective of Parental Involvement in Neonatal Care

In this first thematic line, articles studying the opinion and perspective of parents are presented on their participation in the care of the PN in the NICU. Thus, four research articles were examined in this block, three of them descriptive quantitative studies [29,30,32], and one encompassed within a non-random quantitative study [33]. Two of these studies [32,33], quantitatively descriptive and quantitatively non-random, respectively, were conducted in Sweden and investigated the opinion of parents within the NICU regarding care administration. By contrast, the remaining two articles are descriptive quantitative studies carried out in Latin America [29,30], which assess the effect of parental perception and stress on their participation in the care applied to PNs in the NICU.

Thus, the descriptive quantitative study carried out in Sweden (Uppsala) in 2009 [32] proposed an important initiative, allowing and enhancing the presence of parents, during 24 h periods in these units. Its objective was to know what activities parents did in NICU levels II–III. A sample of 21 PNs and 29 parents (N = 18 women, N = 11 men) was included, and a questionnaire with dichotomous responses was used. Some of the results showed that, of the parents surveyed, approximately 50% (N = 15 parents) fed the PNs by bottle. On the other hand, it was found that about 81% (N = 23 parents) participated in proceedings with healthcare professionals, holding the PN during these proceedings. It was learned that this participation was more common among women (N = 16 women). These findings demonstrate the importance of the role of parents in their children’s care. It was also found that 100% of the parents (N = 29) were able to comfort their child or take their temperature. Finally, 70% (N = 20 parents) were bathing the PN. The results also reflected that it is the men themselves (N = 11) and women (N = 18) who determined their pace of participation in care [32].

On the other hand, the non-random quantitative study, also carried out in Sweden (Gothenburg) in 2017 [33], used the empowerment of parents in the intensive care—neonatology (EMPATHIC-N) questionnaire in 5 NICUs with advanced and high-tech equipment. PNs were included from 24 to 42 weeks gestational age (GA). This questionnaire made it possible to know the opinion of 141 parents (N = 60 women, N = 81 men) on their participation in the administration of care to the PN. It showed a number of items, including the information parents received, or their participation in care. EMPATHIC-N takes into account previous parental experience, and in this study, it was observed that this was slightly higher among men (31%) than among women (28.4%). Similarly, this questionnaire highlighted the importance of the level of studies to integrating the family into the administration of care to the PN. In this sense, women had higher university qualifications (61.7%) (41.4%), which enabled a better understanding and acquisition of knowledge, compared to men, with regard to daily activities. Similarly, about 70% of parents (N = 99) considered that the verbalization of their concerns and the permanence close to their children, during the conduct of procedures, contributed to the reduction of stress and learning of the administration of care to the PN. 

Regarding the two descriptive studies included in this thematic block, they were conducted in Latin American countries (Brazil and Chile). These describe the perception and stress of parents in the administration of PN care in the NICU [29,30]. 

Specifically, the study developed in Brazil (São Paulo) in 2016 [29] sought to evaluate, using measuring instruments such as PCCF-P, PCCF-E, and PSS:NICU, the stress and pre- and post-intervention parental perception related to the involvement of parents in PN care within a neonatal unit when it comes to high-risk patients. The sample was made up of 132 parents (N = 84 women, N = 48 men). The majority group of post-intervention parents ranged in ages between 31 and 45 years (N = 26). In addition, the majority had a mid-level educational training (N = 30), with respect to the full upper level (N = 6). With regard to previous parental experience, it was found that there was a greater inexperience (N = 55) compared to having some previous experience (N = 11). More positive perceptions were shown regarding the collaboration of parents in the realization of PN care (90%) and the support that parents received by the health team (85%). Even parents argued that after the application of the FCCs, they felt like genuine parents (83.3%) rather than visitors during the hospital stay. 

Entering a PN into the NICU is a stressful event for the family. Parents’ expectations are interrupted due to the temporary separation involved in hospitalization. This issue was reflected in the descriptive quantitative study conducted in Chile (Santiago) in 2017 [30]. The purpose of this study was to know the level of stress and the perception of parents regarding their participation in care of a PN in a level III NICU. This study estimated that between 20% and 30% of parents (N = 100) had postpartum depression or feelings of incompetence for care. Additionally, no significant differences were shown between the stress levels experienced by women (N = 57) and those experienced by men (N = 43). The condition of primiparous (61%) or multiparous (39%) did not influence this perception, either. Similarly, the level of university education in women (54.4%) and men (48.8%) was not indicative of any increase in the levels of stress of each parent. It should be noted that in the investigation, one of the issues that most concerned the parents was the inability to protect the PN (N = 59), against the pain caused by the procedures. In addition, in equal measures, men and women suffered from great feelings of impotence, not knowing how to relieve their child. However, without a doubt, the most stressful aspect for the parents (92%) was the alteration of the parental bond. All of these statements reinforce the importance of fostering physical contact between parents and the PN.

### 3.2. Parental Training through the Health Team 

The second line of argument includes articles exploring the training process, which is led by the health team. To this end, three research articles were selected, which are quantitative non-random [33], descriptive quantitative [36], and qualitative [31]. In this case, two of the studies were conducted in Latin American countries (Mexico and Argentina) and investigated the training that parents receive to provide care to their PN within the NICU, which is provided by the healthcare professionals of these units.

Specifically, a descriptive quantitative design study carried out in Mexico (San Luis Potosí) in 2010 [36] sought to understand the scope of care (N = 9 hospitals) in which parents administered and participated in the care of the PN in the NICU (N = 3 level III, N = 3 level II, N = 3 level I). Of the total hospitals included in the study, 55.5% were in the public sphere, and 45.5% were in the private sector. Likewise, it was found that there were no visiting slots in 88.8% of the hospitals, a situation that encouraged greater opportunities for parental collaboration in the administration of care. On the other hand, it is important to note that in 11.2% of public and private hospitals, time restrictions within these units were still in force.

It was shown that the role of nursing is to guide parents in the learning process [36]. Promoting a change in the culture of care requires a transformation in the attitudes of the health team and support of the institutions. In the context of PN care, motivating and encouraging parents to participate in daily routines is a challenge for healthcare professionals [33]. This includes training in basic care, such as teaching mothers to perform bathing of the PN (33.3%), or changing their clothes (55%). Parents were also gradually instructed to care for their PNs in more specific forms of care, such as management of colostomies or oxygen therapy devices (44.4%) [36]. 

In the studio of Gallegos-Martínez et al. [36], it was stated that in severely ill PN cases, continued care at home may be required. Therefore, it is recommended that the parents be trained to be able to provide care at the home level [36]. Having a university education had a positive influence on the administration of care, without forgetting that this situation was also linked to a cultural fact. Therefore, it was found that university graduates represented 61.7% of women, while the percentage among men was 41.4% [33]. It was found that 22.2% of parents were trained during the entire period of admission, scheduling their discharge and planning the acquisition of skills so that they would be ready when they were discharged. By contrast, 55.5% of the parents received training guidelines during the two or three days before the hospital discharge. In an intermediate manner, in only 22.2% of cases was the family trained the week before discharge. In addition, it was demonstrated how the nursing department managed to train parents in care management, strengthening the relationship with the unit’s own team [36].

By contrast, a qualitative study developed in Argentina (Córdoba) in 2018 [31] shows the importance of identifying the care needs of parents of PNs admitted to the NICU. To this end, it conducted in-depth interviews with eight parents (women and men), as it sought to recognize that they can provide care at discharge, thanks to the training they received during their hospital stay. The parents reported having felt supported by the health team, and that the knowledge acquired from the health professionals allowed them to progress and gain autonomy in the care of their children. In addition, they considered that the professionals offered them information in a clear, complete, and simple way, which facilitated their learning. Therefore, this study concluded that orienting attention to the family allowed leading a cultural change, improving the experiences and capacities of the parents in the administration of care to the PN [31].

### 3.3. Benefits of Involving the Family in the Realization of Care

In this third thematic block, research articles are included which try to determine the beneficial effects of incorporating the family in the provision of care to the PN within the NICU. A total of six articles were examined here, three of which were randomized quantitative studies [22,34,37] and two non-randomized quantitative studies [38,39]. There is also one article, included in other categories, which in this case is a descriptive quantitative study [29].

With respect to the non-randomized quantitative studies, two of them [38,39] were conducted in Italy in 2016 and 2017 and assessed the effectiveness of implementing the FCC model of parental involvement in the care of the PN in the NICUs. On the other hand, randomized quantitative studies [22,34,37] have been developed at the international level in Australia, Canada, and India. These studies investigate the impact on parents of promoting their participation in the care of the PN.

Specifically, one of the randomized quantitative studies was developed in Canada (Toronto) in 2013 [34]. Its purpose was to explore the feasibility and results of implementing the FCC model in NICUs. To do so, it had a sample of 42 PNs 35 weeks (N = 28 intervention group, N = 14 control group) and 42 mothers (N = 28 intervention group, N = 14 control group), from which four twin PNs were previously excluded because they presented hemodynamic instability. In order to carry out the study, mothers were asked to stay in the NICU for more than 8 h a day and to attend educational sessions for 3 weeks or even until they were discharged from hospital. Forty percent of the mothers (N = 17) had another child between 2 and 15 years old, which made it difficult for them to remain in the NICU. In terms of educational level, 23.8% (N = 10) had completed secondary education, 14.28% (N = 6) had completed graduate school, and 61.90% (N = 26) were working outside the home, which also posed a barrier to adherence to the study.

A questionnaire (PSS: NICU) was used to determine the level of stress suffered by the mothers whose children were admitted to the NICU. A measurement was carried out in the first week of admission, and another before discharge. The scores in the intervention group (30.6%) and in the control group (32.5%) were similar to those following admission. However, in the intervention group, stress decreased upon discharge (23%), remaining unchanged in the control group (32.5%). This issue reflected the importance of increasing the time spent in attendance, and the involvement of parents in the care within these units [37].

Questionnaires were also used to understand the benefits of FCCs in mothers. A total of 83.33% of mothers (N = 35) pointed out that they had gained self-confidence in the administration of care to the PN, motivated by the change in the relationship with the health equipment. A total of 47.61% of those interviewed (N = 20) revealed that being able to provide care to the PN made them feel safer and calmer, experiencing less stress. The researchers observed that in the PNs of the intervention group, there was a weight gain of 34.5%, compared to 32.2% of the PNs in the control group. Additionally, the rate of breastfeeding was 82.1% in the intervention group, compared to 45.5% in the control group. It is important to note that there was no incidence of nosocomial infection in the intervention group, while in the control group, 9.7% (N = 6) was recorded. Therefore, all these results support the benefits of parental involvement in the care of their children. They even managed to improve the parameters of the PN, and its adaptation to the extrauterine environment, promoting higher rates of breastfeeding, which improves the maternal–subsidiary bond [37].

Another random quantitative study, conducted in India, New Delhi, in 2017 [37], set out to assess the impact of parental involvement on PN care at NICU Level III. The sample was composed of 295 PNs (N = 148 intervention group, N = 147 control group), of which the vast majority had a GA between 35 and 42 weeks. Specifically, it accounted for 76.8% (N = 113) of the sample in the control group, and 81% (N = 120) in the intervention group. Of the family caregivers, 43% were mothers, 37% parents, and 20% grandparents. It was noted that only 2% of them had received a university education. In terms of age, 40% were in the range of 25–35 years. The PNs of the intervention group were cared for by their parents. These were previously trained with audiovisual material and practical demonstrations. Meanwhile, the PNs of the control group received only routine care from nurses and doctors.

The results showed that of the 75 episodes of nosocomial infection produced, 50.66% (N = 38) corresponded to the control group and 49.33% (N = 37) to the intervention group, so there were no significant differences in the occurrence of infections. There was also no disparity in the length of hospital stay, with an average of 11 days for the control group, and 11.5 for the intervention group. However, discrepancies appeared in the mortality data, with the control group having a rate of 8.8% (N = 13), compared to 6.8% (N = 10) of the intervention group. Similarly, the rate of breastfeeding in the control group represented 66.7% (N = 98), compared to 80.4% (N = 119) in the intervention group. These data suggest that although there is no evidence of a significant impact on the incidence of nosocomial infection, the impact of mother participation and preparation can be highlighted in the intervention group’s breastfeeding rates. Therefore, the empowerment and skills acquired by mothers are considered, in this study, to be a cornerstone for the continuation of home care [34].

In Sydney, Australia, a random quantitative study was developed in 2020 [22], which identified the need for parents to participate in PN care, hospitalized in a surgical NICU (Level III). Income and discharge needs were identified through an inventory of neonatal family needs (NFNI) and a social desirability scale (SDS). To carry out the research, a sample consisting of 48 parents (N = 23 intervention group, N = 25 control group) and 48 PNs were available. Regarding these, 69% (N = 33) were born at the full term, and 77% (N = 37) had a weight of more than 2500 g.

The needs detected by the parents were ordered hierarchically according to the importance the parents expressed. Thus, those related to safety were in first place (3.8%), followed by physical proximity (3.6%), together with the information received (3.5%). In last place were those related to the support of the healthcare team (3.1%), and comfort (3%). It was also observed that all the scores increased at the time of discharge from the hospital. Specifically, there was a greater desire to recognize parents’ contribution to the recovery of the PN by 41.7%. This could be related to the age of the parents, since 85% (N = 41) were younger than 40 years old. In addition, 42% (N = 20) of the parents had received university education [22].

The parents showed interest in being able to help with the care of their children (28.8%), a situation that increased the sense of security and control in the administration of care to the PN. Considering that for 60% (N = 28) of the parents, this was their first child, and 96% (N = 46) had no previous experience in the NICU, all of these results suggest that parents want to actively participate in daily care, as well as gaining recognition of their role as caregivers in NICU [22]. This situation was similar to that produced in a study mentioned above [29], where there were more positive perceptions in parents following collaboration in the realization of PN care (90%) and the support received by the health team (85%) [22].

In Italy, two non-random quantitative studies were carried out, specifically in Milan in 2016 [38]. The intention of these studies was to evaluate the effectiveness of an individualized care program, focused on the development of PNs, known as the NIDCAP method, which fostered parental support and participation in care. To do this, they had a sample of 43-32 week PNs (N = 21 intervention group, N = 22 control group) entered into a NICU (Level III), and 33 mothers (N = 17 intervention group, N = 16 control group) who had access to the unit 24 h a day. In the intervention group, the evaluations were made from birth to discharge by two professionals trained in NIDCAP using a neurofunctional evaluation (NFA). In this PN group, only 9.5% (N = 2) suffered moderate deterioration. By contrast, in the control group, which received standard care from a NICU, there was a higher rate, with 38.1% (N = 9) of the PNs.

The involvement of mothers in care was evaluated through a parent support nursing tool (NPST), an issue for which the educational level of parent support was important. It was shown that 50% (N = 10) of the intervention group had an intermediate formation compared to 36.4% (N = 8) of the control group. The NSPT questionnaire was completed by mothers prior to discharge and assessed aspects such as information received, participation in care, or support offered by the health team. It was seen how the mothers of the intervention group gave higher scores. In total, 100% (N = 17) of these mothers were allowed to participate in the care of their children, compared to the mothers of the control group, 40% (N = 6) of whom were involved in the care of their babies. Likewise, 90% (N = 15) of the intervention group felt like true mothers, compared to 33.3% (N = 5) of the control group. This indicates that mothers who participated in the care using the NIDCAP method and were supported by qualified health workers were most involved in the exercise of their maternal role [38]. 

Another non-random quantitative study was developed in Italy (Naples), in 2017 [39]. This study sought to compare the levels of satisfaction and stress suffered by families of PNs admitted to the NICU (Level III). The intervention group applied the FCC model (N = 24 women, N = 24 men, N = 24 PNs), while the control group (N = 24 women, N = 24 men, N = 24 PNs) received traditional care. Parents in the intervention group were allowed to stay in the NICU for 8 h a day, unlike the control group, who only had 1 h per day. A total of 48% (N = 23) of the parents of both groups had completed secondary education.

Upon discharge, both women and men were asked to complete a separate questionnaire to determine the degree of parental satisfaction. This included nine items, such as knowledge, understanding, or collaboration. The results showed that the women and men in the intervention group were more satisfied with both the information received and the relationship established with the health team. Additionally, to measure the stress of parents, a scale (PSS: NICU) was used, which took into account the alteration of the parental role and the difficulties in being able to care for their children. The data showed a statistically significant difference, as the parents in the control group had higher stress averages than those in the intervention group. Within the control group, no differences were found between women and men. All of these issues show the benefits of implementing the FCC model in parents, since it decreases anxiety and increases the security they experience within the NICU. Likewise, for PNs as a whole, the FCC model means that they require less respiratory support, regulate their temperature better, and even promote weight gain [39].

### 3.4. Humanization NICU Care

In the last thematic block, research articles are included which try to enhance the humanization of care, offered to PNs in NICUs, through the participation of parents. This fact is becoming increasingly relevant in the field of neonatal intensive care. Two studies have been selected, which are qualitative [40] and mixed [35].

In 2019, a qualitative study was carried out in Holland (Amsterdam) [40], which proposed a significant change in the infrastructure of the NICU (Level II) to enhance the empowerment of parents in the humanized care of the PN. The study included 53 families. A new architectural design was proposed, and three rooms were merged into one. Single-family rooms were created, which allowed the joint accommodation of the PNs with the parents. As for the characteristics of the rooms, 41.50% (N = 22) of the rooms were for women in the dilation and delivery period, and 33.96% (N = 18) in the postpartum period, accompanied by their PNs. Finally, 24.52% (N = 13) were reserved for the PN, allowing in these rooms cohabitation with both parents during the day, and with one of them during the night. With this structural change, the researchers sought to promote a new paradigm, enhancing respect and dignity, within humanized care and promoting a link between parents and NP, thus facilitating parental participation in care.

Semistructured interviews were conducted which reflected the parents’ desire for good preparation. Parents also expressed the need to have the necessary information before discharge. They also considered it beneficial to have a single room, as well as a joint room for meetings, with all the parents present in the NICU, favoring bonds between them. It is important to highlight the parents’ perceptions, regarding the increase of their autonomy and the empowerment in the care of the PN, in these units. The authors (Stelwagen et al.), concluded that this new distribution seems to promote parental development and the humanization of care [40].

The mixed study included in this integrative review, carried out in Colombia (Cali) in 2016 [35], was intended to evaluate the strategies for the development of the FCC model in NICUs (Level III). To this end, interviews were conducted to learn the perceptions of seven mothers regarding the FCC model. Part of the results obtained were analyzed from a quantitative perspective. It was shown that parents’ access to NICUs increased from 83.3% to 100%. The support that parents received from health professionals increased by 4.2%. Parental involvement in care was approximately 96%. Even communication both at the time of admission and during the hospital stay evolved to 100%. This study reveals that after the application of the FCC model, there are very positive changes in health care, taking into account respect, dignity, exchange of information, participation, and collaboration, which allow evolution toward humanized care.

## 4. Discussion

The revised studies show that family-centered care is a benefit for both parents and the PN. However, it should be noted that the implementation of this model has not been without discussion [32]. It can be said that during the 1980s–1990s, activities related to family care were initiated within th NICU [11]. Today, however, a paradigm shift from family empowerment in these units is being actively enhanced [10,13,18].

It is evident that parents of preterm newborns experience feelings of stress and inability to care, due to the loss of ideal conditions, for the performance of their role as primary caregivers [29,30]. For this reason, the FCC model was developed, which is considered a standard in care, as the family is understood as the main source of support for the PN [29]. In short, parents were integrated and involved in the care of newborns admitted to the NICU [9].

The change in the culture of care involves sharing the role of care between nursing and the family, making the paternalistic vision disappear, where the health team possessed “absolute knowledge”, toward a model of commitment and mutual collaboration. However, sometimes, healthcare professionals tend to underestimate the ability of parents to take care of their PN [16]. An article by Kjellsdotter et al. addresses the perception that nurses have about the involvement of parents in care and notes that they still spend more time in the care of the PN, with regard to integrating the family into daily routines [41]. By contrast, several investigations show that parents feel capable of assuming care, which reflects their aspiration to be able to play their true role in the care of the newborn [30,33]. Therefore, it has been found that the role of the Nurse, at first, entails more intervention time, since the role is to take care of the PN and train the family. Later on, the role of health professionals decreases, since the family becomes the main provider of care [29].

In addition, the interventions made by the parents, in relation to the care of their PN, have been proven to strengthen their confidence and autonomy. They even achieve their empowerment in neonatal care [32]. It is evident in revised research that encouraging parental involvement by healthcare professionals increases parental–filial attachment, which is crucial to the correct physical, mental, emotional, and social development of the PN [33].

To be able to train parents in the care of the PN, it is necessary that the health team be trained and educated in the FCC model [36]. Therefore, the nursing discipline is calling for more training to apply this model, since this group is the main educator, due to the direct and continuous contact with the patient and family [16,41]. However, there are studies that indicate that health professionals have the acquired competence to evaluate the aptitude of parents in the care of the PN [31,33]. In addition, working together allows nurses to know the needs of the family and to focus their efforts where parents do not yet feel prepared [36,42].

The articles agree that nurses provide the necessary support and training, which increases parents’ sense of security and confidence in dealing with the care of their PN [36,43]. It has been confirmed that communication is the basis for promoting an adequate therapeutic relationship between the healthcare team and the family [36]. This enables the acquisition of knowledge and skills necessary for the care of the PN [31].

The researchers note that the nurses, despite perceiving the difficulties involved in the implementation of the FCCs, also appreciate the benefits of family participation in the care of the PN in the NICUs. Among these, the increased rate of breastfeeding stands out, which leads to earlier weight gain in the PN. Similarly, lower rates of nosocomial infection in PNs are envied within NICUs [34,37]. Additionally, a lower mortality rate and neurological involvement has been noted. It has also been proven that parents have lower rates of stress due to the knowledge acquired. In fact, they themselves consider that this has an indirect influence on the growth, development, and recovery of their children [38,39].

In such a specialized and technological environment as the NICUs, humanization as indicated by Acosta-Romo et al. is a challenge [44]. However, other research indicates that today, it is just as important to minimize the emotional impact on the family and the PN as the physical recovery of the latter [45]. The literature reviewed shows that the opening of the doors of the NICU and the reduction of their hours, as well as the involvement of parents in the care of their PN, promotes an evolution toward more humanized care. On the other hand, in a study carried out in Sweden, in which 443 health professionals participated, of whom 372 were nurses, it was found that there is controversy among health professionals regarding this issue, due to the interference caused by the permanent presence of parents during the development of more complex procedures [42].

Are parents’ needs being heard by health professionals, however, and are health professionals being allowed to take an active role in parenting? In this line, it was found that some of the research reviewed (Acosta-Romo et al. in 2017, and Osuna Guerrero et al. in 2018) indicates that in order to offer humanized care, a change in family support policies is necessary [44,45]. Even these statements are reinforced in the study by Hernández et al., which points out that during the process of hospitalization, the involvement of the parents and the establishment of the bond reduces the crying of the PN, compared to those who remain without the presence of the family [35]. This fact of including the family in the NICU is reinforced by the study conducted by Stelwagen in 2020, which states that applying the FCC model guarantees that the PN receives humanized care.

Therefore, in the NICU, concrete measures could be proposed to be developed, in order to contribute to the empowerment of the FCC:

(1) Regarding parental participation within these units, it would be convenient to encourage multidisciplinary clinical sessions, with active participation of both health professionals responsible for formal care and parents;
(2) Likewise, hospital opening hours should be unified during 24 h, and it should even be encouraged that one of the parents spend the night in the NICU with their children;
(3) In order to approach parental training, it would be important to promote learning courses where learning is facilitated from basic to more complex care, delivered by professionals who integrate the team. Such training would bring benefits to the PN, since it would contribute to a joint participation of the health professional and the family, avoiding re-admissions;
(4) The proposal of humanization of the NICU, and the modifications that this entails, must be addressed from the medical and nursing directorates, so that they are heard by the hospital management area.

However, it is true that conceptual changes are still necessary within these units to be able to implement this method in the daily routines within the health services.

## 5. Conclusions

This integrative review represents a resource in the construction of knowledge for the nursing profession. As it is used, it shows the importance of the development of interventions within clinical care practice using the FCC methodology. It considers the PN and the family as a unit to be cared for, facilitating the empowerment of parents in daily care. In addition, it is proven that its application manages to balance the high technology that characterizes the NICU with the most sensitive care, to achieve an appropriate cognitive, sensory, and emotional development of the PN. These aspects are a very positive assessment on the part of families, as their role as a primary caregiver in the care and development of their children is promoted.

The new paradigm that empowers parents in the care carried out in the NICU needs a renewed vision for the healthcare professional because they have a new role as a trainer of the family in the care required by their PN. On the other hand, it is key to highlight the attitude of healthcare professionals in relation to the opening of schedules for 24 h. It should be remembered that until relatively recently, the NICU remained closed, and parents were merely passive visitors. The change promoted by the FCCs enables the joint involvement of healthcare professionals and families in the administration of care to the PN.

The summary and comparative data allow commenting that the humanization of care within the NICU involves an ethical approach, since it allows implementing the suggestions made by families with the aim of improving health care. It promotes a change in the way we care, because according to the WHO, PNs are seen as biopsychosocial beings. It has therefore been seen that the needs of parents are heard by healthcare professionals. It is even valued that this new paradigm is the beginning of the development of a more active role by the parents in the upbringing of their kids. FCC care is becoming more relevant today, as parents are encouraged to participate in NICU care skills.

## 6. Strengths and limitations 

This integrative review reports on one aspect, such as the empowerment of parents through the FCC methodology, which is currently gaining greater relevance, since it encourages respect, dignity, and active participation in the administration of care to the PN in the NICU by the primary caregivers.

Limitations include difficulty in selecting current articles; analysis of the published literature may not have reported all data, or may have selected only data relevant to the selected research question. This integrative review did not have a large number of participants in all studies, but the number of studies that include parents in the sample serves as evidence of the validity of the research.

## Figures and Tables

**Table 1:** Terminology used.

Databases	Combination MeSH Terms
PubMed/Medline	○ Infant newborn OR Child development AND Critical care ○ Empowerment AND Family ○ Caregivers OR Family AND Infant newborn
Cochrane CINHAL	○ Neonatal nurses AND Caregivers AND Professional role ○ Psychological stress AND Family ○ Child development OR Infant newborn AND Breast Feeding
Scopus Google Scholar	○ Caregivers AND Critical care ○ Family AND Neonatal nurses ○ Staff satisfaction AND Family ○ Family AND Infant newborn OR Child development ○ Family AND Empowerment

Source: Own elaboration of the authors.

**Table 2:** Overview of included studies showing active parental involvement in neonatal intensive care unit (NICU) care delivery.

Quantitative Studies							
Author(s), Year and Country	Study Design	Study Purpose	Sample Characteristics	Main Variables	Methodological Quality Level	Results	Limitations
Hedberg et al. (2009) Sweden	Quantitative dichotomous responses	Explore parents’ views on parental performance of care in the NICU.	N = 29 parents: - N = 18 women - N = 11 men - N = 21 PNs (19 N-III parents 10 parents N-II)	Parents: - Gender - No of children - Gestational age (GA) PNs - Postnatal age in the study	80%	Parents can take care of the PN. Support the role of parents as caregivers. Nurse educators.	The sample should be bigger
Martinez et al. (2010) Mexico	Quantitative descriptive	Understand the healthcare environment and the administration of parental care. Parent participation in NICU, at different levels.	N = 9 H: - N = 4 private. - Level I - N = 2 public. - Level III - N = 3 mixed. 1 Level III 2 Level II	- Level of care. - Hospital birth rate. - NICU characteristics. - Human resources. - Parental involvement - Post-discharge follow-up	60%	Encourage parent participation. Implementation of the FCC philosophy. Healthcare training and parent care training.	Enlarge sample size. Evaluate infrastructure, equipment, organization.
O’Brien et al. (2013) Canada	Quantitative cohort analysis	Explore the feasibility of implementing the FCC care model. IN NICU promote maternal development: attention to PN.	N = 42 PNs 4 twin PNs were excluded (N =14 PN G. Control) N = 42 mothers (N = 14 mothers G. Control)	Rn: - GA PN - Birth weight - Apgar 1’ and 5’ - Oxygen days - Administration of vasoactive agents and caffeine Mothers: - Marital Status - Age - No of children 2–15 years - Distance from the hospital - Educational level	60%	FCC model is feasible and safe. It improves maternal care and PN results.	Use critical incidence reports only to monitor security. Non-representative, non-generalizable parents
Sannino et al. (2016) Italy	Quantitative non-randomized control single center	Evaluate NIDCAP effectiveness mother care participation PN.	N = 43 PN (32 GE): - N = 21 G. Intervention - N = 22 G. Control N = 33 mothers. - N = 17 G. Intervention - N = 16 G. Control	PN: - GA PN - Birth weight Mothers: - Maternal age - Educational level Questionnaire: - Parent Support - Quality care	60%	NIDCAP effective participation of mother in care of PN improves neurofunctional development. Mothers in NIDCAP group more involved.	Small sample size. No group randomization. Bias of the population studied, only one center
Simphronio et al. (2016) Brazil	Quantitative quasi-experimental	To evaluate the effects of FCC implementation on perception and parental stress on caring capacity.	N = 132 parents of PN (N = 66 phase prior to intervention, N = 66 after intervention).	Parents: - Sociodemographic profile - Distance from the hospital - Hospitalization experiences - Social Support Neonates: - Length of stay - Age - Diagnosis	100%	Improved parental perception in FCC in terms of respect, collaboration and support in the post-intervention phase. Greater satisfaction, increased capacity to care for children. Less parental stress and anxiety after intervention	The study investigated two measures (perception and stress). Short-term evaluation.
By Bernardo et al. (2017) Italy	Quantitative prospective non-randomized cohort	Compare levels of satisfaction and stress, participation and care: - Parents group FCC. - Non-FCC parents.	G. FCC: - N = 24 parents - N = 24 mothers - N = 24 PN G. NO FCC: - N = 24 parents - N = 24 mothers - N = 24 PN	Parents: - Nationality - Age - Educational level Rn: - GA PN - Apgar 1’-5 - Length of stay	60%	FCC Group: higher satisfaction, lower stress level when participating in care. Family integration model advantages, future need for trials.	Small sample size. Selected population of PN disease Qx. Non-randomization. Do not distinguish between procedures that are a source of stress for parents.
Ottosson et al. (2017) Sweden	Quantitative multiple regression analysis	Identify process of care components. Vision parents participation care.	N = 141 parents of NICU children. - N = 60 men - N = 81 women	Characteristics of parents: - University Education - Previous parental experience Questionnaire EMPATHIC-N): - Information for parents, treatment and care. - Parent participation. - Professional attitude.	80%	Strong points of PN: better interaction with breastfeeding and caring. Professional hands-on involvement: facilitates parent participation. Important contact nurse continuity.	No random sample: generalization of results questioned.
Palma et al. (2017) Chile	Quantitative cross-sectional description	Knowing stress levels and parental perceptions of participating PN in NICU care.	N = 100 parents (N = 43 men, N = 57 women) N = 59 RN.	Parents: - Average age - Level of studies - Place of residence Mother: - Parity. - Multiple pregnancy. - Pregnancy complications. - Previous abortions. - Type of delivery	100%	Support and parent education, allows them to cope with stress. Encourage care and practices that promote parent–PN bonding.	Not analyze other factors that can influence stress, such as mental health, social network, personality.
Verma et al. (2017) India	Quantitative randomized controlled trial	Evaluate impact of parent involvement in care of PN.	N = 295 PN NICU: G.Control=147 G.Interven=148 Family: - 37% parents - 43% mothers - 20% grandparents	- Average weight - GA PN - Gender - Type of delivery - Interventions made by parents	80%	G. Intervention, better preparation good home transition. Decreases hospital stay. Parent empowerment: cornerstone of the continuum of care	Study low power to detect differences. No evaluation of long-term results.
Govindaswamy et al. (2020) Australia	Quantitative prospective cohort	Identify needs for parental involvement in NICU care	N = 48 parents of PN (N = 23 G. Intervention N = 25 G. Control) N = 48 PN.	Characteristics of parents: - Age group: or 18–36 (30) or 36–40 (11) or 40 (7) - First child: or Yes (28) o No (20) - Previous NICU experience: or Yes (2) o No (46) Characteristics PN: - Gender: ○ Male (28) or Female (20) - Gestational age: or 28–34 (2) or 34–37 (13) or 37(33) - Birth weight: or 1500 (1) or 1501–2500 (10) or 2501 (37)	60%	Parents G. Intervention need to actively participate in PN care, recognize caregiving role. FCC Model meeting parent needs.	Difficult to evaluate sample representativeness and generalization of results. Includes only parents who can read and write in English. Limited sampling method.
**Qualitative Studies**							
Campo et al. (2018) Argentina	Qualitative phenomenological paradigm	Identify parenting needs. Pick up parental care granted to PN upon discharge.	N = 8 parents children admitted to NICU.	- Emotional support - Coordination and integration of care. - Information, education, family participation. - Physical comfort, support for daily activities.	100%	Parents argue nursing: teaches care, family empowerment Communication improves bonding. Open NICU guides and trains parents in care.	Data are collected until they provide information relevant to the study.
Stelwagen et al. (2019) Holland	Qualitative descriptive	Describes transition from traditional NICU design to new infrastructure that enables parent empowerment	N = 53 families	Room design: - Patient type. - Sanitary equipment. - Hospitalization/High - Joint accommodation - No of meters	60%	Implementation of infrastructure for neonatal care, facilitates parent empowerment. Requires willingness to change.	Minimum description of the implementation mechanism and its cost.
**Mixed Studies**							
Hernandez et al. (2016) Colombia	Quantitative descriptive and qualitative analysis	Evaluate strategies for developing PN and FCC in NICU	N = 7 mothers	- Current problems in care, barriers to FCC implementation. - FCC knowledge and perceptions. - Unity environment, parent involvement, interaction with staff. - Changes in neonatal practice	80%	Healthcare workers: Encourages humanized care in NICUs. Educates the family in PN care, with mutual communication and training. Parents need more communication, participation, and increased visiting hours. The changes to promote FCC obtained positive results in the short term.	Personal change during study, obstacle to continuous reflection. Changes in care practice occurred over a short period of time.

## List of Abbreviations

WHO	World Health Organization
PN	Preterm newborn
NICU	Neonatal Intensive Care Unit
FCC	Family Centered Care
NIDCAP	Newborn Individualized Developmental Care and Assessment Program
GA	Gestational Age
(PCCF-P) Brazilian version	Patient and Family-Parent Focused Care Perception
(PCCF-E) Brazilian version	Patient and Family-Team Focused Care Perception
PSS: NICU	Parental Stress Scale: Neonatal Intensive Care Unit
